# In Situ EBSD Study of Aluminum After Embrittlement by Gallium

**DOI:** 10.3390/ma18051026

**Published:** 2025-02-26

**Authors:** Kaikai Cai, Shuo Wang, Daixin Zhang, Haiyun Feng, Pu Song, Hongwei Hu

**Affiliations:** Xi’an Modern Chemistry Research Institute, Xi’an 710065, China; ckkhhh2000@163.com (K.C.); 19338004697@163.com (S.W.); 17343397424@163.com (D.Z.); dahai99-2005@163.com (H.F.); hhw505@163.com (H.H.)

**Keywords:** liquid metal embrittlement, in situ EBSD, grain boundary penetration mechanism, fracture analysis, microscopic morphology, dislocation motion

## Abstract

Liquid metal embrittlement is a phenomenon in which the mechanical properties of a metallic material are significantly reduced after contact with liquid metal, and the microscopic mechanism of this phenomenon is still controversial. The grain boundary penetration mechanism has recently been widely recognized, but the theory is still deficient. To refine the theory of grain boundary penetration, in this paper, the liquid metal embrittlement mechanism of aluminum by gallium is obtained by in situ EBSD, combining it with the fracture morphology features and comparing the differences of the microscopic feature changes and the crack evolution process during the in situ tensile process of embrittled and untreated aluminum specimens. The results show that the fracture elongation of aluminum decreased by 60% after being embrittled by liquid gallium at 80 °C for 40 min, and the gallium atoms entering the aluminum interior decreased the grain boundary cohesion while promoting dislocation emission. Combining the experimental results and previous studies, we divide the fracture of aluminum after liquid metal embrittlement into three stages, namely, the grain boundary penetration stage, the local fracture stage, and the integral failure stage.

## 1. Introduction

Liquid metal embrittlement (LME) is an event in which a metallic material’s mechanical properties are drastically diminished upon contact with a liquid metal. This phenomenon is primarily characterized by a loss of tensile strength and fracture toughness. As early as the 1920s, embrittlement of brass after exposure to mercury was reported [1]. It has been discovered that some liquid metals, such as Ga, Hg, In, Zn, Pb, Bi, and others, can cause varying degrees of embrittlement in metals, including in copper alloys, steel, and aluminum alloys [2,3,4]. Unlike other forms of failure in metals, liquid metal embrittlement has a remarkable suddenness and specificity. Suddenness is demonstrated by the extremely short time required for LME to occur, with solid metals becoming significantly embrittled in less than a few hours of contact with the liquid metal, e.g., the 7075 aluminum alloy loses about 60% of its strength after 30 min of contact with gallium at 75 °C [5]. Liquid metal embrittlement is sometimes known as catastrophic damage since there are no symptoms when it happens [6]. Specificity is demonstrated by the fact that liquid metal embrittlement occurs only in specific metal couples [7], e.g., mercury embrittles aluminum but not titanium.

Research on the LME was first based on the safety needs of the energy industry, since mercury can cause the LME of aluminum products and has led to serious safety incidents and economic losses. The primary cause of the 1973 severe catastrophe at the Skikda Liquefied Natural Gas (LNG) plant in Algeria was determined to be mercury corrosion embrittlement, which failed the aluminum heat exchanger [8]. The two successive shutdowns of the LNG plant belonging to Hairan Gaoxin Energy Co., Ltd. in Haikou, China, in 2006~2007, were also caused by mercury corrosion embrittlement leading to leakage in the aluminum alloy tubes [9]. Martensitic and austenitic steels, which are used as cladding materials for nuclear fusion reactors, undergo LME with the coolant of the nuclear reactor (lead-bismuth eutectic) [10,11,12,13]. Advanced high-strength steels for automotive applications undergo LME during resistance spot welding [14,15,16], thus LME impedes the development of both the nuclear energy field and the automotive industry. Electron backscatter diffraction (EBSD) is a technique that uses diffracted electron beams to characterize the grain boundaries, phase boundaries, grain orientation, weaving, and strain of a sample. It operates by focusing an accelerated electron beams at the sample, resulting in backscattered electrons diffracted from the sample surface. These electrons include crystallographic information about the surface. This allows one to ascertain the properties of each grain in the sample. The in situ EBSD technique is a variation of the standard EBSD that uses extra benches to regulate experimental parameters like temperature, stress, or magnetic field. This allows EBSD to be observed in a wider range of environments. This technique is frequently applied to metal fracture analysis [17,18,19]. In their investigation of experimental metrics of powder layer quality during selective laser sintering, Lupo et al. [20] introduced an image-processing analysis method that converts a series of microscopic images into layer surfaces along the gray-scale distribution. The gray level profiles were further analyzed with a wavelet analysis tool, and the major features of the wavelet powder spectral density were used to calculate specific indicators of the powder layer quality. The technique introduces a new way of analyzing microstructural interactions in critical situations. In recent years, with the wide application of liquid metals in medical devices, battery energy, 3D printing, computing, and other fields [21,22,23,24], the LME phenomenon has been gradually emphasized by various industries. It has been demonstrated that liquid metal embrittlement is a complicated multifactorial linked phenomenon influenced by several variables, including embrittlement temperature, exposure time, alloy composition, surface condition, grain size, and force application [25]. There is currently no definitive conclusion on the micro-mechanism of LME, even though a thorough study of it is essential to comprehending its laws and avoiding it [26,27,28,29]. A large number of experiments in recent years have shown that the grain boundary penetration mechanism may be the most reasonable explanation for LME [30,31,32], with the main content being that when the liquid metal comes into contact with the solid metal, the penetration of the liquid metal atoms into the grain boundary of the solid metal reduces intergranular cohesion, resulting in metal embrittlement. The theory can qualitatively explain most of the phenomena of LME, but there are still deficiencies. According to the theory, the fracture mode of LME should be dominated by intergranular fracture; nevertheless, the fracture mode of LME is typically a combination of intergranular fracture, transgranular fracture, and cleavage fracture with several modes of fracture [33].

This paper investigates the changes in fracture modes of metals resulting from LME and aims to complete the grain boundary penetration mechanism. For this study, aluminum and liquid gallium were used as experimental materials, and in situ tensile EBSD was used to characterize the mechanical properties and observe the microstructure of the specimens. To obtain appropriate specimens with embrittlement treatment, we devised three specific embrittlement conditions based on prior research. Among these, the mechanical properties of specimens embrittled at 80 °C for 40 min exhibited the most significant differences compared to untreated specimens. By comparing the crack evolution and microstructural changes between the embrittled specimens (80 °C for 40 min) and untreated specimens during the tensile process, we can identify the shifts in the fracture mode of the aluminum specimens before and after LME occurrence. This further elucidates the microscopic mechanism underlying liquid metal embrittlement.

## 2. Materials and Methods

### 2.1. Materials Selection

The solid metal used in this experiment is cast aluminum (Al 99.80%) (provided by Xincheng Aluminum Co., Ltd., in Shanghai, China), and its main components are shown in Table 1. The embrittler is liquid gallium (99.9%) (provided by Huatai Metal Materials Technology Co., Ltd., in Dongguan, China). The main reasons for using cast aluminum and gallium are as follows: 1. The Al/Ga system is capable of significant liquid metal embrittlement [34], and liquid gallium can cause significant embrittlement of aluminum even in the absence of stress. 2. Impurity elements and the grain shape are known to affect liquid metal embrittlement [35], whereas cast aluminum has fewer impurities, and the grains are generalized as unrolled equiaxial crystals. 3. Aluminum has good toughness, and the change in fracture behavior before and after embrittlement will be more pronounced in pure aluminum compared to aluminum alloys such as 7075.

### 2.2. Experimental Design

The primary goal of this experiment is to investigate the changes in the microstructure and fracture behavior of aluminum before and after the occurrence of LME of the metal and then to determine the microscopic mechanism of liquid metal embrittlement, so aluminum specimens without embrittlement as well as those with obvious embrittlement are required. Embrittlement temperature is an important factor influencing the embrittlement effect. According to a study by Bosch et al. [36,37], there is a temperature ductile groove for liquid metal embrittlement. This suggests that the mechanical characteristics of the metal first deteriorate and then recover their previous state as the embrittlement temperature rises. The temperature at which liquid metal embrittlement occurs is frequently higher than the melting point of the embrittlement agent, whereas gallium’s melting point is approximately 30 °C. Aluminum products are usually used in daily life at service temperatures of 100 °C or less. To create an apparent distinction between the mechanical properties of the embrittled and untreated specimens, the experimental temperatures were set at 40, 60, and 80 °C. Furthermore, prolonged contact time between aluminum and gallium (several hours) might cause a material loss on the specimen’s surface as well as significant embrittlement, increasing the specimen’s risk of fracture during microstress. To ensure the embrittlement of the specimens and decrease the possibility of inadvertent breakage, the embrittlement duration was set to 40 min, and the experimental conditions were created, as shown in Table 2.

### 2.3. Sample Preparation

**Figure 1:**
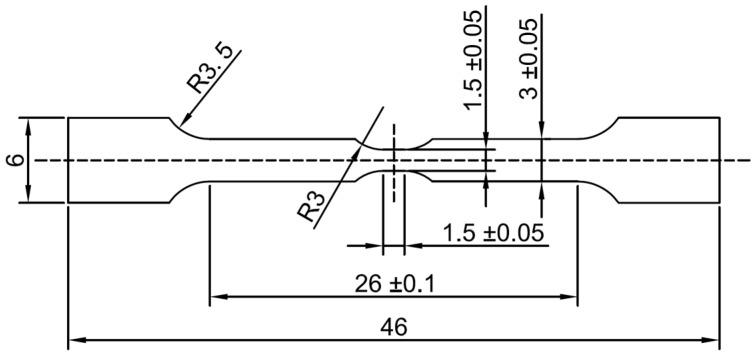
Experimental specimen machining diagram. The specimen thickness is 1 mm, and the unit in the figure is mm.

The aluminum ingot needs to be homogenized because of its irregular interior microstructure. The aluminum ingot was cut into 1 × 8 × 14 cm^3^ plates and placed in a muffle furnace for 10 h. The temperature was kept at 600 °C, and the pressure was atmospheric. Then the plates were taken out and cooled in the air. After homogenization, the aluminum plate was processed by wire-cutting (medium-wire walking on the upper and lower surfaces and fast-wire walking on the sides) to produce the samples shown in Figure 1, with a sample thickness of 1 mm, and all the samples were taken from the same direction of the same aluminum plate.

**Figure 2:**
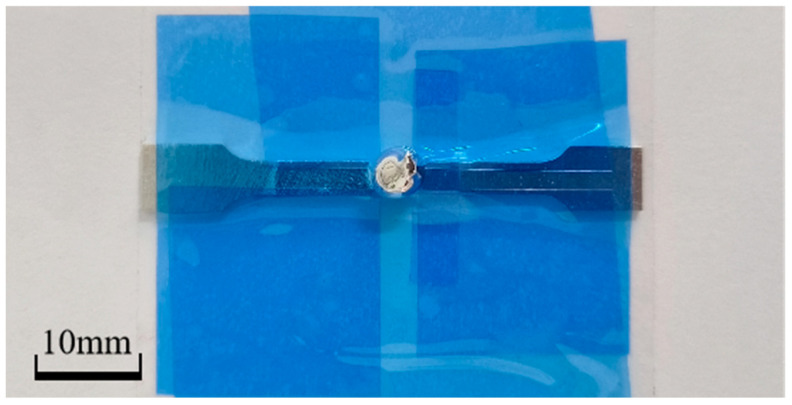
Schematic of specimen embrittlement (liquid gallium is covered in the scalar segment).

For the preparation of specimens, this paper used the method of electrolytic polishing followed by one-sided embrittlement, which can effectively prevent the embrittled metal specimen from being damaged during the preparation process while obtaining a smooth surface. This is considering the liquid metal will erode the specimen’s surface after embrittlement and cause damage, but the EBSD requires a high surface finish of the specimen. The samples were sanded smooth using 240, 800, 1500, 2000, 3000, 5000, and 7000 grit sandpaper in turn, then polished to remove scratches with an aluminum oxide polishing solution containing 2.5 μm and 0.5 μm particles. The specimens were then electrolytically polished using a 1:9 perchloric acid alcohol solution (provided by PCL Labeling Technology Co., Ltd., in Chengdu, China), with the specimens placed at the anode; the polishing voltage was 20 V, and the polishing time was 20 s. As indicated in Figure 2, the next phase of the process was as follows: paste a blue metallographic protective coating on the polished side of the specimen, keeping it facing down; attach the specimen to the cardboard using blue tape, exposing only the scalar segments on the unpolished side; use a syringe to cover about 0.1 mL of liquid gallium in the specimen’s non-polished surface’s scalar segment, and then put the specimen into the thermostat for embrittlement. Experimental conditions are shown in Table 2. After 40 min, the specimen was removed, the liquid gallium on its surface was washed off, and anhydrous ethanol was used to clean it.

**Figure 3:**
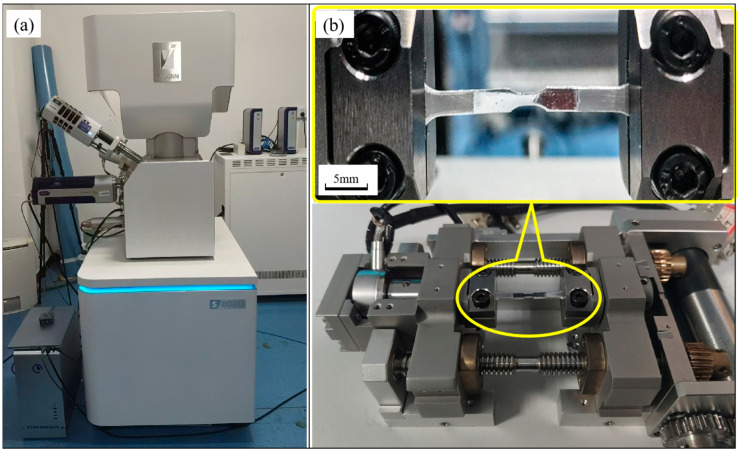
In situ EBSD equipment: (**a**) TESCAN S8000 scanning electron microscope; (**b**) specimen stretching station.

The polished surface of the embrittled specimen was observed using an in situ EBSD apparatus as shown in Figure 3. The experiments were conducted using a scanning electron microscope (SEM) model TESCAN S8000 (manufactured by TESCAN in Brno, Czech Republic), as shown in Figure 3a, which had an accelerating voltage of 50 V–30 kV and a maximum magnification of 2,000,000 times. It was also equipped with an Oxford Symmetry S1 EBSD detector (manufactured by Oxford Instruments in Abingdon, UK), which had a calibration rate of over 3000 points per second. The observation range was initially chosen from the specimen’s scalar segment in the SEM mode after the specimen was first mounted on the in situ stretching stage, as illustrated in Figure 3b. Then, using a scanning step of 4 μm and an exposure length of 10 ms, the EBSD data were obtained. After completing the first data collection, the specimen was stretched at a speed of 0.5 μm/s. The stretching was continued until the sample yielded. The EBSD data acquisition was repeated under the same conditions, followed by in situ stretching at 0.5 μm/s. Once the micro-morphology of the sample changed significantly (e.g., the appearance of dense slip lines, emerging cracks, concentrated deformation, etc.), the stretching was suspended, and EBSD data acquisition was carried out. Stretching was continued after completing the data acquisition, and so on until the sample was pulled apart.

An SEM5000 scanning electron microscope (manufactured by Guoyi Quantum Technology Co., Ltd., in Hefei, China.) was used to observe the fracture micro-morphology of the drawn specimen, while an energy dispersive spectrometer was used to analyze the elemental distribution of the specimen fracture.

## 3. Results

### 3.1. Mechanical Test Results

**Figure 4:**
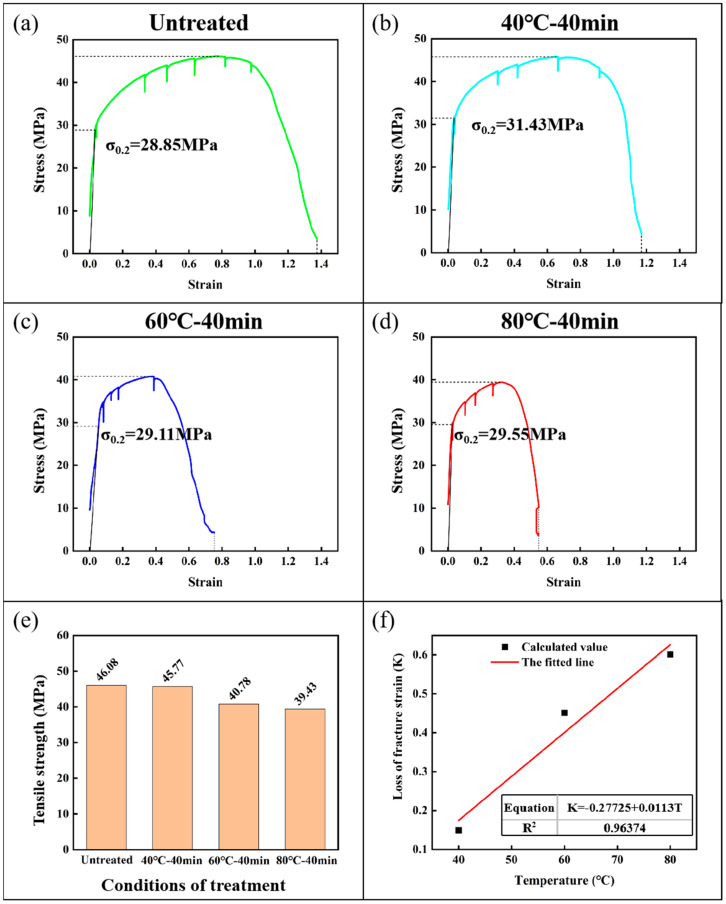
Changes in the mechanical properties of specimens under different experimental conditions: (**a**) stress–strain curve of untreated specimens; (**b**) stress–strain curve of embrittled specimens at 40 °C—40 min; (**c**) stress–strain curve of embrittled specimens at 60 °C—40 min; (**d**) stress–strain curve of embrittled specimens at 80 °C—40 min; (**e**) tensile strength of specimens under different experimental conditions; (**f**) graph of the value of strain loss at fracture K of embrittled specimens versus embrittlement temperature, the equations and R^2^ values of the fitted line are in the figure.

As shown in Figure 4, the mechanical properties of aluminum specimens under different embrittlement conditions were obtained by in situ stretching, and Figure 4a–d shows the stress–strain curves of the specimens under different experimental conditions. There are inverted peaks in the stress–strain curve as stretching has to be paused during the experiment to obtain EBSD data. These stress–strain curves show that aluminum specimens have no obvious yielding stage in the tensile process, so the stress value that produces 0.2% residual deformation is taken as its yield strength. The diagrams demonstrate that the aluminum’s yield strength varies very little before and after embrittlement, essentially remaining at 30 MPa. However, the strain at fracture reduction is very evident before and after the embrittlement of the specimen; the strain at fracture for the untreated aluminum specimen is 1.37, and, after 80 °C—40 min embrittlement treatment, it is only 0.55. According to Figure 4e, which displays the specimens’ tensile strength under various embrittlement conditions, the embrittled specimens’ tensile strength will be marginally lower than that of the untreated specimens, and it will decrease as the embrittlement temperature rises. K represents the strain loss at the fracture of the specimen, and the variation curve with the embrittlement temperature is presented in Figure 4f.

K = (ε_u_ − ε_e_)/ε_u_ (1)

where ε_u_ denotes the fracture strain of the untreated specimen and ε_e_ is the fracture strain of the embrittled specimen. According to Figure 4f, K and the embrittlement temperature have a positive correlation; the higher the embrittlement temperature, the more pronounced the embrittlement effect and the more severe the strain loss at specimen fracture. This is essentially consistent with other research [36,37]. This is because, as the temperature increases, more gallium atoms penetrate the Al/Al matrix, leading to more severe embrittlement.

### 3.2. Microcosmic Observation

**Figure 5:**
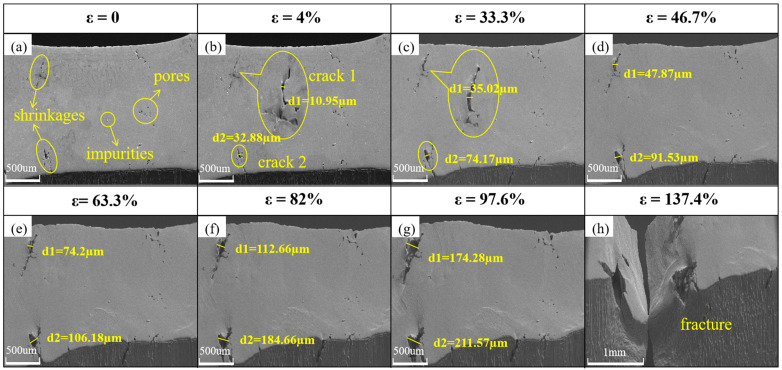
Changes in surface micromorphology of untreated specimens during in situ stretching. Crack widths in the figure are averages calculated from ten measurements. (**a**–**h**) SEM images of untreated specimens at different strains, respectively.

Figure 5a–h shows the surface micromorphology of the untreated specimens during in situ stretching with a stretching speed of 5 μm/s. The microscopic morphology of the specimen before loading is depicted in Figure 5a. It is evident that the specimen’s surface still has noticeable flaws following grinding and polishing, including impurity spots, pores, and shrinkage holes created during the cooling process of aluminum. When the specimen has just yielded, as shown in Figure 5b, its strain is 4%. Under stress, the specimen is uniformly stretched, and the imperfections gradually develop into cracks. Cracks 1 and 2 are located in the upper and lower left corners of the field of view, respectively, with a width of 10.95 μm and 32.88 μm. Figure 5a,b shows the elastic deformation stage, where the bonds between aluminum atoms lengthen. If loading is stopped, the specimen will return to its initial state; if loading is continued after yield, some of the specimen’s atoms’ bonds will break and the atoms will begin to slip; the macroscopic manifestation of this is that the specimen will begin to undergo plastic deformation [38]. The specimen in Figure 5c has a strain of 33.3%, cracks 1 and 2 have widths of 35.02 μm and 74.17 μm, respectively, and the microcracks on the specimen’s side have begun to climb. In Figure 5d, the specimen exhibits localized inhomogeneous deformation when the strain reaches 46.7%. However, the stress is still gradually increasing at this point, and cracks 1 and 2 are growing, with the widths of cracks 1 and 2 reaching 47.87 μm and 91.53 μm, respectively. The specimen’s surface appears to have clear slip bands, and flotation starts to appear on the surface to coordinate the deformation. In Figure 5f, the specimen’s strain hits 82%, crack 1’s width increases to 112.66 μm, and crack 2’s breadth rises to 184.66 μm. The specimen exhibits clear concentrated deformation as cracks 1 and 2 widen toward one another. Dense slip bands are also present close to the crack, suggesting that stress concentrations exist there. The strengthening stage of the specimen is shown in Figure 5b–f. During this stage, the specimen is uniformly elongated under stress, numerous dislocations begin to slip, entangle, and accumulate at the grain boundaries and near the cracks, and the specimen experiences deformation strengthening as the stress increases steadily. With the further increase in strain, the crack expands rapidly, and the specimen starts to undergo concentrated deformation, but the rate of stress increase slows down until the strain reaches about 80%, and the stress reaches a maximum value of 46 MPa. Subsequently, necking occurs in the specimen, the real stress area of the specimen becomes smaller, the stress also begins to decline rapidly, and the deformation enters the fracture stage. The specimen’s strain reaches 97.6% in Figure 5g, and cracks 1 and 2’s widths increase to 174.28 μm and 211.57 μm, respectively. As shown in Figure 5h, the specimen eventually fractures along the plane where cracks 1 and 2 are located after the strain reaches 137.4%. The specimen’s fracture stage is depicted in Figure 5f–h. After the specimen has undergone central deformation, the strain is primarily concentrated in the plane containing cracks 1 and 2, and the specimen eventually fractures close to the cracks. Calculated independently, the average crack expansion velocities of specimens’ cracks 1 and 2 at the strengthening stage (Figure 5b–f) were v_1_ = 4.3 × 10^−5^ m/s and v_2_ = 6.4 × 10^−5^ m/s.

**Figure 6:**
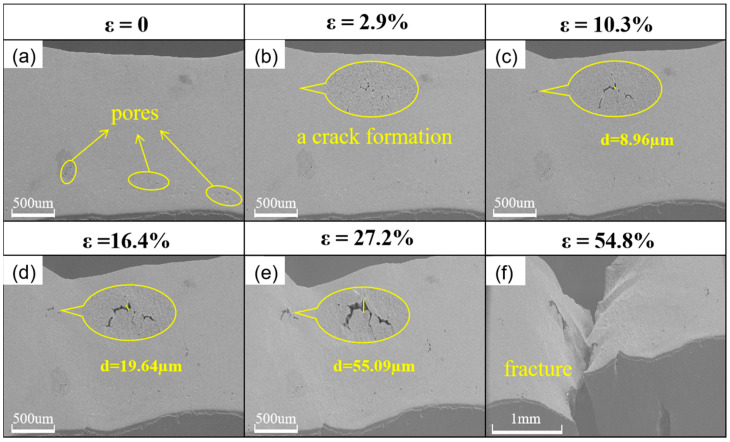
Surface micromorphological changes of embrittled specimens during in situ stretching at 80 °C—40 min. Crack widths in the figure are averages calculated from ten measurements. (**a**–**f**) SEM images of 80 °C—40 min embrittled specimens at different strains, respectively.

The specimens’ surface micromorphologies under 80 °C—40 min embrittlement conditions during in situ stretching at a speed of 5 μm/s are displayed in Figure 6a–f. Figure 6a shows a small number of pores on the surface of the specimen. The specimen yielded at a strain of 2.9%, as illustrated in Figure 6b, at which point microcracks appeared on the specimen’s surface. The crack is nucleated from the impurity point, as can be seen by comparing with Figure 6a; otherwise, the specimen shows no discernible alteration. The specimen begins to deform non-homogeneously in Figure 6c when the strain reaches 10.3%, at which point the microcrack’s breadth grows to 8.96 μm, but the stress continues to rise continuously. The specimen deforms considerably in the direction perpendicular to the observation surface when the strain reaches 16.4%, and the crack width increases to 19.64 μm. Upon reaching 27.2% strain, the specimen experiences significant deformation perpendicular to the observation surface, the crack width increases to 55.09 μm, and visible slip bands emerge on the specimen’s surface. The specimen broke along the severe deformation at a strain of 54.8%, as seen in Figure 6f. The specimen’s average crack expansion velocity during the strengthening stage (Figure 6c–e) was calculated to be v = 8.7 × 10^−5^ m/s.

### 3.3. Substructure and Dislocation Motion

**Figure 7:**
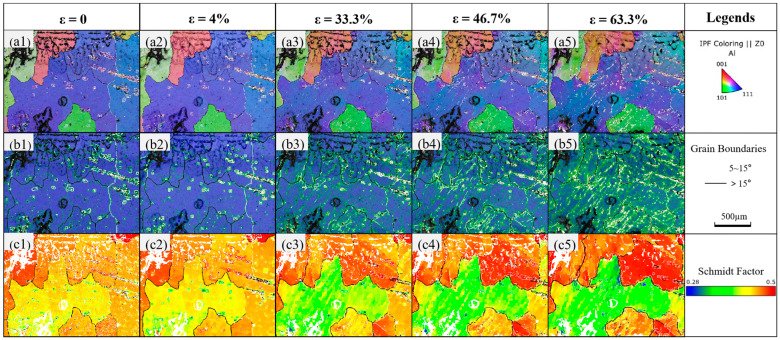
IPF, KAM, and SF figures of untreated specimens at different strains: (**a1**–**a5**) are inverse pole figures; (**b1**–**b5**) are kernel average misorientation maps; (**c1**–**c5**) are Schmidt factor maps.

Electron Backscatter Diffraction (EBSD) is a technique that uses diffracted electron beams to analyze the crystallographic characteristics of samples, by which dislocation distribution, substructure information, and statistical information on grain boundary features of polycrystalline metals can be obtained. Detailed information on the microstructure of the specimen at different strains can be obtained by an EBSD scanner equipped with an in situ stretching stage. In this experiment, the scanning step was kept at 4 μm, and the exposure time was 10 ms during the EBSD data acquisition. Figure 7 shows the inverse pole figures (IPF, (a1)~(a5)), kernel average misorientation maps (KAM, (b1)~(b5)), and Schmidt factor maps (SF, (c1)~(c5)), for the untreated specimen. The black grain boundaries in the figures indicate high-angle grain boundaries (>15°), while the white grain boundaries indicate low-angle grain boundaries (5–15°). Different colors in the inverse pole figures represent grains with different orientations, and the darker color in the Schmidt factor maps indicates a larger Schmidt factor. According to the IPF, the specimen was initially organized as an equiaxed crystal with a diameter of several hundred microns. It also has additional defects, and the atomic orientation next to the flaws is angled concerning the other atoms, creating a subgranular border. KAM maps can be used to characterize the geometric dislocation density or the amount of substructure by calculating the angle of local dislocation in the grains [39]. It can be seen from the KAM maps that a small amount of dislocation exists within the grains at a strain of 0, which is mainly concentrated at the grain boundaries and subgrain boundaries. The grain gradually elongates as the strain grows, and the dislocation concentration within the grain does not change much when the strain reaches yield. This suggests that the atomic key’s elongation is the primary cause of the deformation at this stage, while interatomic slip is reduced. The specimen reaches the deformation enhancement stage when the strain keeps increasing. The dislocation concentration rises sharply and is mostly found in the grain and subgrain boundaries, with numerous subgrain boundaries proliferating under the influence of stress. Grain color variations in the IPF figures show changes in grain orientation during the stretching process, and dislocations fill the grain interiors as the strain is increased further. Coordinated deformation also occurs between the grains. The densest crystallographic direction of atomic arrangement is typically the direction of atomic slip; therefore, crystals typically use dense rows of surfaces as slip surfaces. The {111} crystal plane dominates the relatively fixed slip surface of aluminum, a face-centered cubic crystal type, and the <110> crystal direction is typically the slip direction [40]. When stretching, the Schmidt factor maps of the specimen on the {111} <110> slip system are displayed in Figure 7(c1–c5). The crystal is prone to slip in that direction if the Schmidt factor is higher, and the red area of the plot is more prone to slip.

**Figure 8:**
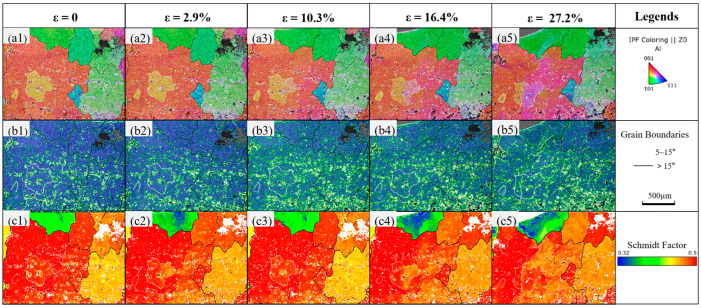
IPF, KAM, and SF figures of 80 °C—40 min embrittled specimens at different strains: (**a1**–**a5**) are inverse pole figures; (**b1**–**b5**) are kernel average misorientation maps; (**c1**–**c5**) are Schmidt factor maps.

Figure 8 shows the inverse pole figures (IPF, (a1)~(a5)), kernel average misorientation maps (KAM, (b1)~(b5)), and Schmidt factor maps (SF, (c1)~(c5)), for the embrittled specimen at 80 °C—40 min. The IPF figures demonstrate that, during in situ stretching, the embrittled specimens’ grain orientation also changed. Alongside Figure 6, it is also discovered that, in contrast to the untreated specimen, the crack of the embrittled specimen appears at grain boundaries and spreads out along them as strain increases. The embrittled specimen before stretching has more subgrain boundaries, and some dislocations are entangled around these subgrain boundaries and grain boundaries, according to a comparison of the KAM maps of the specimens before and after embrittlement. There is minimal atomic slide in the embrittled specimen prior to yield, as evidenced by the dislocation density not changing significantly as the strain increases and the specimen reaches yield. The embrittled specimen reaches the enhanced stage as the strain increases. At this stage, atomic slip emits a lot of dislocations and grows a lot of substructures, which cannot cross the grain boundaries and eventually build up close to the substructures and grain boundaries, causing concentrations of microstress. Compared to the untreated specimens at the same strain, the embrittled specimens had a substantially higher dislocation density and a greater number of substructures. The SF maps of the embrittled specimens on the {111} <110> slip system is shown in Figure 8(c1–c5), and they are compared to the SF maps of the untreated specimens. The Schmidt factor of the embrittled specimens is found to be much larger than that of the untreated specimens, indicating that embrittlement facilitates the opening of the aluminum’s {111}<110> slip system.

### 3.4. Micro-Morphologic Observation of the Fracture

**Figure 9:**
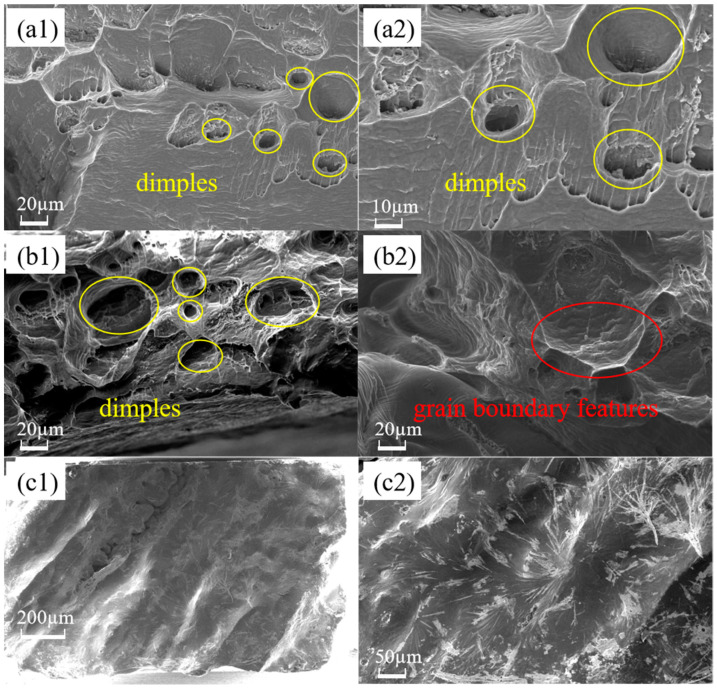
Fracture microscopic morphology of specimens under different experimental conditions: (**a1**,**a2**) are the fracture morphology of untreated specimens; (**b1**,**b2**) are the fracture morphology of specimens embrittled at 80 °C—40 min; and (**c1**,**c2**) are the fracture morphology of specimens embrittled at 80 °C—40 min with accidental fracture.

**Figure 10:**
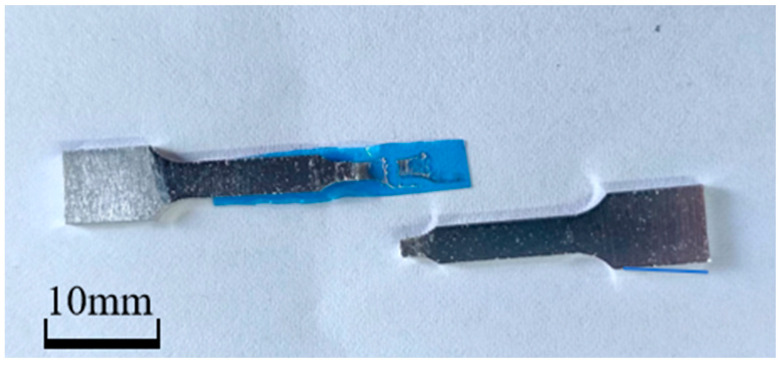
Accidental fracture of a specimen after embrittlement at 80 °C for 40 min.

**Figure 11:**
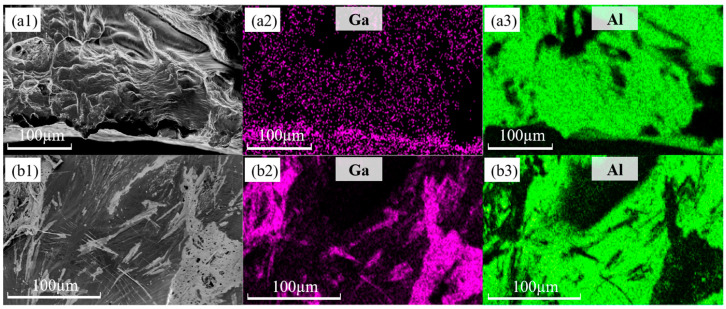
Elemental distribution of specimens fracture surface: (**a1**) fracture topography of 80 °C—40 min embrittled specimen; (**a2**) Ga elemental distribution of 80 °C—40 min embrittled specimen; (**a3**) Al elemental distribution of 80 °C—40 min embrittled specimen; (**b1**) fracture morphology of accidentally fractured specimen after 80 °C—40 min embrittlement; (**b2**) Ga elemental distribution of fracture of accidentally fractured specimen after 80 °C—40 min embrittlement; (**b3**) Al elemental distribution of accidentally fractured specimen after 80 °C—40 min embrittlement).

Fracture scanning of the pulled specimen reveals the change in the fracture pattern. The fracture microscopic morphology of the untreated specimen is depicted in Figure 9(a1,a2), where it is evident that the fracture is a distinct microporous aggregation type ductile fracture with unequal dimple diameters and white tearing ribs. Because aluminum is so durable, the dimples’ sizes vary from a dozen microns to tens of microns. Additionally, the fracture is distributed on some larger voids, which are formed in the casting process of aluminum pores, shrinkages, and other discontinuous defects in the stress growth formation. In the tensile process of microporous nucleation, growth, connection, stress concentration at these defects, cracks in the crystal formation, and continuous expansion ultimately lead to the specimen’s transgranular fracture. Figure 9(b1,b2) shows the microscopic morphology of the fracture of the embrittled specimen, and many dimples and tear ribs can still be seen in Figure 9(b1), but obvious grain boundary features are found in Figure 9(b2). This shows that the aluminum in gallium embrittlement experienced a partial first intergranular fracture in response to the external force, while the unbroken area retained good plasticity. However, stress concentration occurred, which ultimately resulted in a significant reduction in the specimen’s fracture strain, and the fracture exhibits a combination of intergranular and transgranular fracture morphology. The investigations also revealed another intriguing phenomenon: as illustrated in Figure 10, a specimen fractured during the evacuation of surplus liquid gallium following 40 min of embrittlement at 80 °C. It occurs owing to the needle unintentionally scraping the oxide layer on the specimen’s surface when adding liquid gallium, which caused the specimen to become severely embrittled by gallium [41]. The embrittled specimen had very low strength and broke under microstress. The same fracture scanning was carried out on the specimen with the accidental fracture, and the results are shown in Figure 9(c1,c2), which show that the fracture of this specimen is smooth and flat, covered with grayish-white irregular patterns, showing obvious cleavage fracture characteristics. An energy dispersive spectrometer was used to analyze the fracture surface elements of the specimen embrittled at 80 °C for 40 min, and the specimen fractured accidentally. As shown in Figure 11, the results show that the fracture of the two specimens has an obvious distribution of gallium, in which the distribution of gallium in the specimen of the accidental fracture is more obvious, and the grayish-white irregular pattern observed in the microscopic morphology is gallium, but the distribution of gallium elements in the two does not show obvious regularity.

## 4. Discussion

By comparing the stress–strain curves of the specimens under different embrittlement conditions, it is found that there is no significant difference in the performance of the specimens in the elastic deformation stage before and after embrittlement, but the enhanced deformation stage of the specimens after embrittlement is shortened. As an example, consider the 80 °C—40 min embrittled specimen. The untreated specimen will only experience a stress reduction when the strain reaches 80%, whereas the 80 °C—40 min embrittled specimen will begin to experience a stress reduction when the strain reaches 27.2%. Ultimately, the fracture strain of the embrittled specimen is only 40% of the untreated specimen, and the tensile strength also clearly decreases. Furthermore, it was found that, at higher embrittlement temperatures, the LME impact was more pronounced. Micro-morphological observation and the comparison of aluminum specimens before and after embrittlement in the fracture process reveal that, despite a significant reduction in plastic deformation capacity, the specimen after embrittlement still exhibits distinctive toughness fracture characteristics (clear necking). Additionally, there is a significant deformation of the specimen perpendicular to the direction of the observation surface following embrittlement, which may be the result of inhomogeneous deformation caused by the partial fracture of the specimen inside the embrittled specimen. The influences on LME are multiple and difficult to decouple, making it difficult to study them quantitatively. We have used the method of dimensional analysis to quantitatively study the embrittlement rules of liquid metal, and the specific results can be found in the Supplementary Materials.

Observation of the specimen fracture by SEM revealed that gallium atoms penetrated the aluminum matrix. Rostoker et al. [26] proposed that the adsorption of liquid metal on solid metal surfaces would lower the solid metals’ surface energy and, consequently, their fracture strength. However, Yamaguchi et al. [7] demonstrated through simulations that the specificity of the LME was controlled by atomic penetration and that the surface adsorption energy of liquid metal atoms on solid metals did not correlate with the specificity of the LME. Pereiro et al. [42] conducted a more thorough investigation of the gallium penetration process in aluminum substrates. Gallium penetrates aluminum grain boundaries at a rate of roughly 25 μm/s, according to synchrotron radiation X-ray microradiography. This process can be spontaneous since interfacial energy decreases during the penetration process. Pereiro et al. classified the penetration of gallium into aluminum into three stages: (1) the propagation and simultaneous thickening of the liquid film; (2) thickening discontinuities with fast propagation rates; and (3) the saturation of the layer thickness. In their study, Kelley et al. [43] provided a method for calculating the metal bonding energy, and it was discovered that the Al/Ga bonding energy was 76.8% lower than the Al/Al bonding energy, which is the primary cause of aluminum. This is the main reason why cohesiveness at the grain boundaries is declining. By simulating the penetration of gallium along the aluminum grain boundaries using a molecular dynamics approach, Nam et al. [44] discovered that gallium penetrates along the aluminum grain boundaries and produces stresses that are high enough to cause dislocations at the grain boundaries. Once the dislocations are formed, they will “climb” along the grain boundaries at an almost constant rate, and when the first dislocation is far away from the nucleation region, other dislocations will be formed and “climb” along the grain boundaries. Nam’s theory was confirmed by comparing the KAM maps of the aluminum specimens before and after embrittlement (Figure 7(b1) and Figure 8(b1)). The dislocation density of the embrittled aluminum specimens was much higher and concentrated close to the grain boundaries before stress was applied to the aluminum specimens, suggesting that gallium infiltration encouraged the nucleation of dislocations at the grain boundaries. As the strain increased, the dislocation density also increased, the dislocations became entangled with each other and difficult to move, and the overall plasticity of the specimen was seriously reduced, and the embrittled specimen became more prone to stress concentration after applying stress, and the speed of crack expansion was also significantly accelerated. This is consistent with the calculation results of this paper.

**Figure 12:**
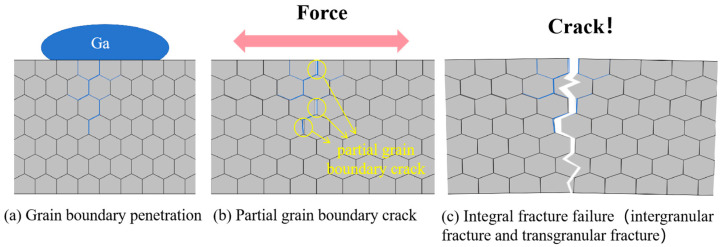
Schematic representation of the fracture of aluminum after embrittlement by gallium: (**a**) gallium penetration at aluminum grain boundaries; (**b**) local grain boundary cracking; (**c**) integral fracture failure.

Based on the experimental results and combined with the views of previous authors, we believe that the liquid metal embrittlement of aluminum by gallium is divided into three stages (as shown in Figure 12): The first stage is the penetration of gallium at aluminum grain boundaries. Without external force, gallium atoms penetrate the aluminum matrix, and most of the gallium atoms are concentrated near the aluminum grain boundaries, which leads to a decrease in the cohesion of the aluminum grain boundaries. A small portion of gallium atoms enter the crystal, replacing the aluminum atom positions, and the Ga/Al and Ga/Ga bonds are weaker than the Al/Al bonds [44], which makes the specimen more susceptible to atomic slip and dislocation emission after embrittlement under external forces. The velocity of gallium penetration into aluminum depends on factors such as temperature, the duration of exposure to gallium, the condition of the metal surface, and the specificity of the embrittlement system [22]. The second stage is the partial cracking of the embrittled aluminum, in which those grain boundaries with severely reduced cohesion under the action of external forces undergo partial fracture to form intergranular fracture characteristics, while other regions still maintain good plasticity and continue to undergo plastic deformation. However, if the specimen is severely embrittled, excessive Ga atoms in the crystal instead of Al atoms will lead to the specimen’s cleavage fracture. The third stage is the overall failure of the specimen. After the occurrence of the local fracture of the specimen forces concentration, local crack expansion speeds up, and ultimately crack destabilization leads to specimen fracture.

## 5. Conclusions

In this paper, the following conclusions were obtained from in situ EBSD as well as fracture surface scanning experiments on aluminum specimens before and after being embrittled by gallium:

1. Liquid gallium metal will cause the serious LME of aluminum, mainly manifested in the reduction in elongation at the break. In a certain temperature range, when the embrittlement time of the specimens is the same, the higher the embrittlement temperature, the more the embrittlement effect is obvious. In aluminum specimens at 80 °C and 40 min of liquid gallium embrittlement, the fracture strain fell to 40% of the untreated specimens.
2. After embrittlement, the plastic deformation ability of the specimen is greatly reduced, and cracks are easily generated at grain boundaries and grow along the grain boundaries under the action of external forces, and the crack expansion rate of the embrittled specimen is significantly higher than that of the untreated specimen.
3. Under stress, the penetration of liquid metal gallium atoms can promote dislocation emission and substructure formation in aluminum.
4. Aluminum specimens under the action of LME can be divided into three stages of tensile fracture, which are as follows: gallium atoms in the penetration of aluminum grain boundaries, local intergranular cracking under the action of external forces, and the integral failure of the specimen, and, ultimately, the fracture presents a complex mixture of fracture characteristics. However, excessive gallium atoms in the aluminum matrix will lead to the specimen exhibiting the second stage of the direct occurrence of cleavage fracture.
5. The microscopic mechanisms and macroscopic regulations of LME have essentially been clarified after almost a century of research. The LME phenomenon for common binary metal systems is also more clearly recognized. However, there is a general difficulty in studying the LME phenomenon in poly-alloy systems and conducting quantitative studies of LME because of the excessive number and coupling of factors affecting liquid metal embrittlement. A more comprehensive examination of LME from the standpoint of atomic energy can be carried out in the future by applying the first nature principle. On the other hand, a large number of experiments can be carried out under different conditions for certain typical combinations of metal materials and embrittlement agents with mature preparation processes (e.g., 7075 aluminum alloy and gallium) to obtain relatively accurate empirical formulas for LME under specific conditions.

## Figures and Tables

**Table 1:** Element composition of aluminum ingots.

Elements	Fe	Si	V	Zn	Al
wt%	0.11%	0.04%	0.02%	0.01%	≥99.8%

wt% indicates mass percentage.

**Table 2:** Specimen embrittlement conditions.

Specimen Number	Embrittlement Liquid	Embrittlement Temperature/°C	Embrittlement Time/min
1	/	/	/
2	Ga	40	40
3	Ga	60	40
4	Ga	80	40

Specimen No. 1 is an untreated specimen.

## Data Availability

The original contributions presented in the study are included in the article, further inquiries can be directed to the corresponding author.

## Supplementary Materials

The following supporting information can be downloaded at: https://www.mdpi.com/article/10.3390/ma18051026/s1.
