# Supplementary material for: In Situ EBSD Study of Aluminum After Embrittlement by Gallium
Source: Materials (Basel). 2025 Feb 26;18(5):1026. doi: 10.3390/ma18051026 (PMC11901249; doi:10.3390/ma18051026)
Supplement: Supplementary file 1 [file materials-18-01026-s001.zip › materials-3436079-supplementary.pdf]

Supplementary:

The tensile strength of the aluminum alloy material is selected as the defining parameter for embrittlement, and the correlation between the influencing factors and the defining parameter is established using the metal type, ambient temperature, and embrittlement time as the key influencing factors. Multiple physical quantities related to the tensile strength of the metal after embrittlement of the liquid metal are used to represent this coefficient, assuming that only the selected physical quantities affect the tensile strength. By unifying the dimensions of these physical quantities through physical relational equations, an exponential value for the effect of the chosen physical quantity on tensile strength can be obtained. This gives the mathematical relationship between the tensile strength and each physical quantity under the assumption, and thus the relationship between the main characterizing parameter and the key influencing factors. Liquid metal embrittlement is an extremely complex problem, and the following assumptions need to be made to study it using the dimensional analysis method:

(1) Liquid metal embrittlement is the phenomenon of the weakening of material properties based on intergranular atomic encroachment; the movement of liquid metal atoms between metal crystals is regarded as diffusion, and the motion of the atoms is thermal; the system of unitary sub-molecules, with an advective degree of freedom of 3N-3 in the center-of-mass coordinate system, has the following total kinetic energy:

$$E_K=\sum_i \frac{1}{2} m_i v_i^2 = \frac{3N-3}{2} k_B T, \qquad (S\,1)$$

where Ek denotes the total kinetic energy, mi denotes the mass of the atom, v_i denotes the velocity of the atom, 3N-3 denotes the translational degrees of freedom, k_B denotes the Boltzmann constant, and T denotes the thermodynamic temperature.

(2) Liquid metal is a monatomic molecular system, and the interactions between liquid metal atoms are not considered; they are independent individuals both in intergranular motion and in bonding with metallic materials.

(3) The size of the bonding energy between liquid metal and solid metal directly affects the embrittlement results, and the calculation method of Kelley et al. [45] is considered here.

(4) According to the existing experimental studies, the liquid metal embrittlement of many metals on the temperature dependence of the "ductile groove" effect at lower temperatures (higher than the melting point of the liquid metal and much lower than the melting point of the solid metal) shows a better dependence on the temperature once the temperature is too high, and many even began to appear strengthened. The present study is only for the temperature range of good dependence on the embrittlement effect. The study in this manuscript only focuses on the temperature range where the embrittlement effect has a good temperature dependence.

(5) Liquid metal embrittlement timescale varies according to the type of metal and different environments; short instances can be completed in a few minutes, long instances can last for months and demonstrate the embrittlement of the embrittlement (the embrittlement effect), i.e., the time taken to enhance the tendency to weaken until completely independent of time. This manuscript is only for the embrittlement that has just begun after the embrittlement of a period of the embrittlement effect with an obvious dependence on the period.

(6) The value investigated is the reduction in tensile strength of the solid metal, i.e., the difference between the tensile strength before embrittlement and the tensile strength after embrittlement.

(7) To make the results meaningful, the zero value of the temperature starting point is the melting point of the liquid metal.

The relevant physical quantities selected according to the above assumptions are shown in Table S1.

Table S1: Physical quantities related to the embrittlement law of liquid metals.

Parameter type	Physical quantity	Unit.	Dimensional analysis
Target parameter	Tensile strength σ	Pa	$L^{-1}M^{1}T^{-2}\theta^{0}$
Geometric size	Specimen diameter d	m	$L^{1}M^{0}T^{0}\theta^{0}$
	Specimen length l	m	$L^{1}M^{0}T^{0}\theta^{0}$
Liquid metal parameters	Density ϱ_l	kg/m^3	$L^{-3}M^{1}T^{0}\theta^{0}$
	Electronic excitation energy E_{pl}	eV	$L^{2}M^{1}T^{-2}\theta^{0}$
	Mass M	kg	$L^{0}M^{1}T^{0}\theta^{0}$
Solid metal parameters	Density ϱ_s	kg/m^3	$L^{-3}M^{1}T^{0}\theta^{0}$
	Electronic excitation energy E_{ps}	eV	$L^{2}M^{1}T^{-2}\theta^{0}$
	Atomization energy E_a	J	$L^{2}M^{1}T^{-2}\theta^{0}$
Thermodynamic parameters	Temperature T	K	$L^{0}M^{0}T^{0}\theta^{1}$
	Thermodynamic entropy increase rate $S^{®}$	J/Ks	$L^{2}M^{1}T^{-3}\theta^{1}$
	Diffusion velocity v	m/s	$L^{0}M^{-\frac{1}{2}}T^{-\frac{1}{2}}\theta^{0}$
Other parameters	Time t	s	$L^{0}M^{0}T^{1}\theta^{0}$

The target parameter tensile strength is a function of the other variables, so it can be written as follows:

$$\sigma=F\{d, l, \varrho_l, E_{pl}, m, \varrho_s, E_{ps}, E_a,, T, S', v, t\} \qquad (S\,2)$$

According to the principle of dimension consistency, there must be

$$[\sigma]=[F\{d, l, \varrho_l, E_{pl}, m, \varrho_s, E_{ps}, E_a, T, S', v, t\}]=[L^{-1}M^{1}T^{-2}] \qquad (S\,3)$$

By the Π theorem, the dimensionless relation is established as follows:

$$\Pi\frac{\sigma}{Pa}=F'\left\{\frac{d}{m},\frac{l}{m},\frac{\varrho_l}{\frac{kg}{m^3}},\frac{E_{pl}}{\frac{kg\cdot m^2}{s^2}},\frac{m}{kg},\frac{\varrho_s}{\frac{kg}{m^3}},\frac{E_{ps}}{\frac{kg\cdot m^2}{s^2}},\frac{E_a}{\frac{kg\cdot m^2}{s^2}},\frac{T}{K},\frac{S'}{\frac{kg\cdot m^2}{s^3\cdot K}},\frac{v}{\frac{m}{s}},\frac{t}{s}\right\} \qquad (S\,4)$$

The expression is simplified by taking the dimensionless quantity as the similar parameter of the independent variable. Since the principle of dimension consistency only guarantees the unity of the dimension and does not distinguish which physical quantity contributes to that dimension, all physical quantities with the same dimension are grouped into one, e.g., E_{pl}、E_{ps} and E_a in equations are both energies, which are

treated as a single dimension in the calculation. The basic principle to be followed in selecting the quantities to be calculated is not to select base quantities, such as individual lengths, times, etc., while selecting quantities that cover all the base quantities involved, with no fewer than two physical quantities containing each of the base quantities, or else there will be a zero solution, and, finally, to make sure that the quantities selected are independent of each other, and to avoid selecting physical quantities that are linear in the form of the quantities.

Four physical quantities are selected here to establish simplified dimensionless relational equations:

$$\prod \sigma = F'(E, \varrho, S', v) \qquad (S5)$$

Establish a computational equation based on the Π theorem:

$$[\sigma] = \prod_1^4 [M_i]^{\xi_i} = [E]^{\alpha}[\varrho]^{\beta}[S']^{\gamma}[v]^{\delta} = [L^{-1}M^1T^{-2}] \qquad (S6)$$

According to the principle of dimensional consistency, the following is obtained for each of the four basic dimensions:

$$\begin{cases} [L^2]^{\alpha}\cdot[L^{-3}]^{\beta}\cdot[L^2]^{\gamma}\cdot[L^0]^{\delta}=[L]^{-1} \\ [M^1]^{\alpha}\cdot[M^1]^{\beta}\cdot[M^1]^{\gamma}\cdot\left[M^{-\frac{1}{2}}\right]^{\delta}=[M]^1 \\ [T^{-2}]^{\alpha}\cdot[T^0]^{\beta}\cdot[T^{-3}]^{\gamma}\cdot[T^0]^{\delta}=[T]^{-2} \\ [\theta^0]^{\alpha}\cdot[\theta^0]^{\beta}\cdot[\theta^{-1}]^{\gamma}\cdot\left[\theta^{\frac{1}{2}}\right]^{\delta}=[\theta]^0 \end{cases} \qquad (S7)$$

Obtaining:

$$\begin{bmatrix} 2 & -3 & 2 & 0 \\ 1 & 1 & 1 & -0.5 \\ -2 & 0 & -3 & 0 \\ 0 & 0 & -1 & 0.5 \end{bmatrix} \cdot \begin{bmatrix} \alpha \\ \beta \\ \gamma \\ \delta \end{bmatrix} = \begin{bmatrix} -1 \\ 1 \\ -2 \\ 0 \end{bmatrix} \qquad (S8)$$

Solution:

$$\begin{cases} \alpha=\frac{2}{11} \\ \beta=\frac{9}{11} \\ \gamma=\frac{6}{11} \\ \delta=\frac{12}{11} \end{cases} \qquad (S9)$$

That is, $[\sigma]=\prod_1^4[M_i]^{\xi_i}=[E]^{\alpha}[\varrho]^{\beta}[S']^{\gamma}[v]^{\delta}$ can be used with the dimensionless coefficient λ to express it as a physical relationship with the following scale: $\sigma=\lambda\cdot E^{\frac{2}{11}}\cdot\varrho^{\frac{9}{11}}\cdot dS^{\frac{6}{11}}\cdot v^{\frac{12}{11}}$. According to the definition of σ to calculate the index of the single basic measure T, θ , by the average kinetic energy of atomic motion $E_K=\sum_i\frac{1}{2}m_iv_i^2=\frac{3N-3}{2}k_BT$ can be obtained $v_i\propto T^{\frac{1}{2}}$, according to the results of the calculation you obtain $\sigma\propto v_i^{\frac{12}{11}}$, it is then easy to obtain $\sigma\propto T^{\frac{6}{11}}$. Similarly, according to the entropy growth rate $S'=\frac{dS}{dt}=\frac{dQ}{dt\cdot T}$, compared with the characterization of the energy of the measure of $S'=\frac{E}{t\cdot T}$, $\sigma\propto S'^{\frac{6}{11}}=\left(\frac{E}{t\cdot T}\right)^{\frac{6}{11}}=\frac{E^{\frac{2}{11}}}{t\cdot T^x}$, it is easy to obtain $x=\frac{1}{3}$, because of $\sigma\propto E^{\frac{2}{11}}$, the following can be obtained: $\sigma\propto t^{\frac{6}{11}\times\frac{1}{3}}=t^{\frac{2}{11}}$.

So far, through the method of dimension analysis to establish the liquid metal

embrittlement characterization parameter σ under the premise of meeting the assumptions with the temperature T, time t as the relationship between the equation is

$$\Delta\sigma=\xi\cdot T^{\frac{6}{11}}\cdot t^{\frac{2}{11}}, \quad \text{(S 10)}$$

where the parameter ξ characterizes the strength of the ability of liquid gallium to embrittle aluminum alloy.

On the one hand, due to the large number of assumptions made before the formula was established, and on the other hand, because inter-atomic interactions and the surface state of the contact surfaces were not taken into account before the study, it resulted in the fact that the above results were only valid for the liquid metal embrittlement of gallium and aluminum alloys for a certain range of temperatures and periods.
